# Endometriotic Mass After Hysterectomy in a 61 Year Old Post-menopausal Woman: A Case Report and Update

**DOI:** 10.3389/fsurg.2019.00014

**Published:** 2019-05-10

**Authors:** Marie Pierre Mathey, Jean Bouquet de Jolinière, Attila Major, Francois Pugin, Etienne Monnard, M. Fiche, Daniel Sandmeier, Fathi Khomsi, Anis Feki

**Affiliations:** ^1^Département de Chirurgie Gynécologique et Oncologique, Hôpital Cantonal, Fribourg, Switzerland; ^2^Département de Chirurgie Générale, Hôpital Cantonal, Fribourg, Switzerland; ^3^Département de Radiologie et Radiologie Interventionnelle, Hôpital Cantonal, Fribourg, Switzerland; ^4^Institut de Pathologie, Centre Hospitalier Universitaire Vaudois (CHUV), Lausanne, Switzerland

**Keywords:** endometriosis, malignant transformation, mullerian adenofibroma, post-menopausal woman, hysterectomy

## Abstract

Endometriosis is a common, hormone-dependent gynecologic disease. Undiagnosed in large proportion of women, managing therapies depend on the impact of quality of life and includes hormonal treatment and pelvic surgery. Less likely endometriosis can occur in post-menopausal women. Malignant transformation of endometriosis is a rare but well-described process, most of time occurring in the ovary, and justifies the practitioner not to underestimate this pathology. We present a case of a 61 year old woman with a symptomatic endometriotic pelvic mass, status post hysterectomy, with no history of endometriosis diagnosed beforehand.

## Introduction

Endometriosis is a benign, hormone-dependent and inflammatory disease. Described for the first time in 1925, it is characterized by the presence of endometrial tissue outside of the uterine cavity, occurring most commonly in fertile women (1, 2). The main symptoms are infertility and pain. The exact prevalence of this disease remains unknown, but is thought to be around 6–10%, with only 2–5% being diagnosed after menopause (3). Endometriosis can occur in post-menopausal women with or without hormonal replacement therapy which indicates the complex mechanism of perpetuation of this pathology. Hard to diagnose because of the absence of pathognomonic symptoms or biomarkers, endometriosis in only confirmed by biopsy during laparoscopy. Magnetic resonance imaging is a non-invasive option but with week sensitivity and no exclusion value (4).

Although endometriosis is a benign proliferative disease; it does share common characteristics with neoplastic processes [inflammatory state, invasion of adjacent tissues, induction of angiogenesis, and resistance to apoptosis (5)]. Malignant transformation of endometriosis is a well-documented though rare phenomenon that occurs most commonly in the ovaries (6, 7). However, it seems that endometriosis is not associated with an increased risk of cancer (8).

Sarcomas are a rare and heterogeneous group of malignant tumors of mesenchymal origin, affecting principally soft tissues (80%) but also bones (20%) (9). The heterogeneity of sarcomas, with regard to molecular genesis or histology pattern, makes its diagnosis very challenging.

This paper presents the case of a 61 year old woman, status post hysterectomy, presenting a pelvic mass with vaginal bleeding and diagnosed with deep pelvic endometriosis.

## Case Study

A post-menopausal 61 year-old 2G1P woman presented to the Gynecology Obstetric Hospital in Freiburg, Switzerland, in January 2018 for severe metrorrhagia without abdominal pain. Medical history included high blood pressure and moderate dyslipidemia. The patient was under hormone replacement therapy (HRT). In her past medical history, the patient had a cesarean and a total hysterectomy by laparotomy in 2000, without ovariectomy, for menometrorrhagia (no morcellation needed). The pathological report of the hysterectomy specimen showed a focal hyperplasia of the endometrium without atypia and a submucosal leimoyoma.

**Figure 1:**
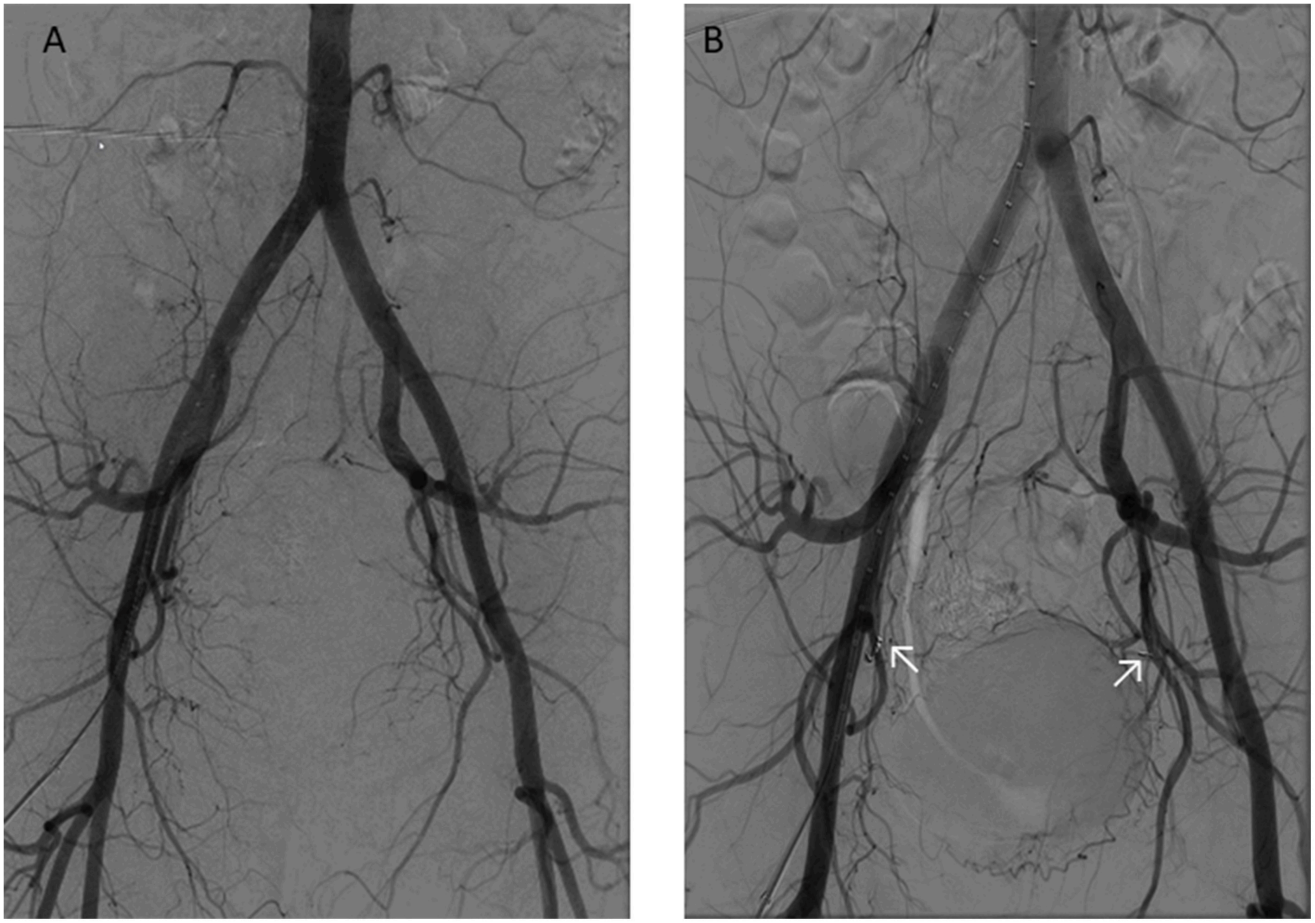
The importance of the bleeding led to an embolization of the vaginal artery with coil fixing. **(A)** Before embolization. **(B)** After embolization.

During her visit, the medical examination showed a normal abdominal status and the vaginal palpation done by the junior doctor seemed normal. Nevertheless, the speculum examination revealed a budding mass of the vaginal dome. A vaginal ultrasound showed a heterogeneous highly vascularized lesion above the vaginal dome measuring 53 × 66 mm. The ultrasound showed a normal right ovary, the left ovary was not seen. There was no free liquid in the peritoneal cavity. We partially stopped the bleeding by dabbing with silver nitrate but as the bleeding was not totally stopped, we performed an embolization of the right and left vaginal arteries with coils (Figure 1).

Further anamnesis revealed that this mass had been known since 2013 and discovered after a CT scan exam and ultrasound performed because of vaginal bleeding. At that time, it measured 45 mm in its greatest dimension. The patient had decided not to pursue further investigations as tumor markers (Ca-125, Ca 19-9, Ca 15-3, alphaFP) were normal and for personal reasons.

**Figure 2:**
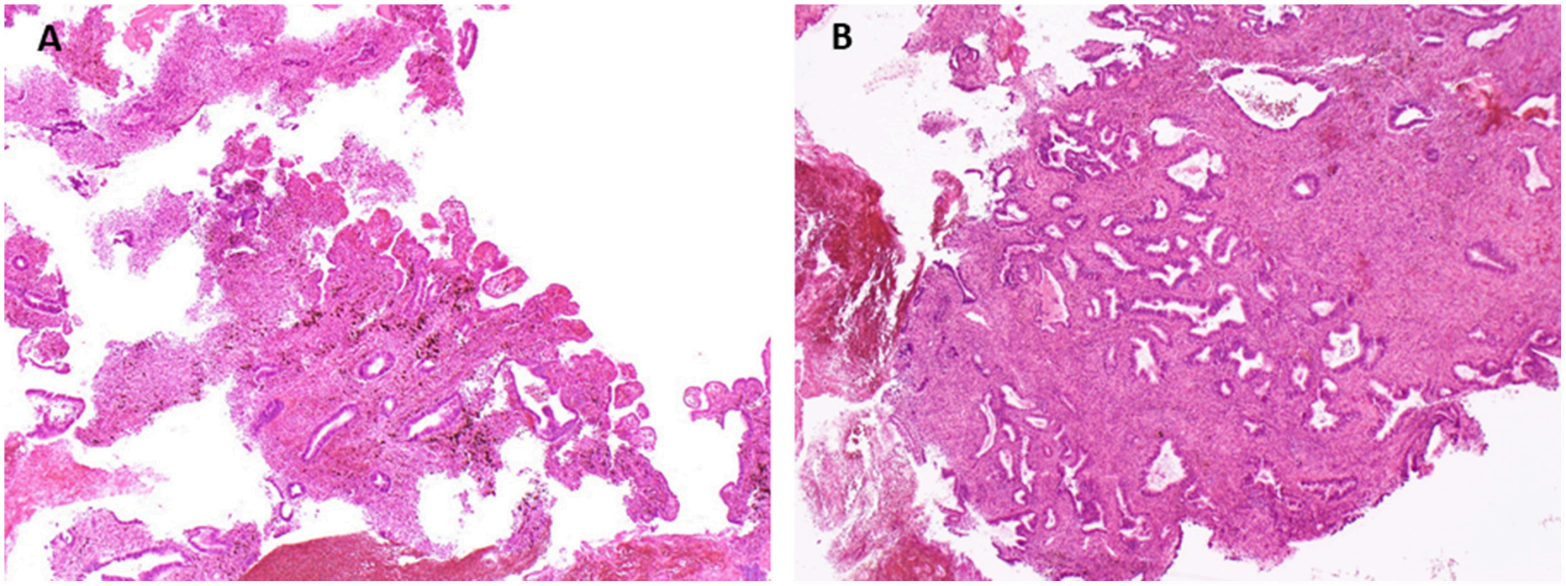
Vaginal biopsies. The tissue transmitted for pathological analysis corresponds to endometrium, with numerous glands slightly irregular in size and shape lined by cylindrical cells without nuclear atypia, and densely cellular stroma with signs of ancient bleeding, rare mitotic figures, and no cytologic atypia. **(A)** Hemalun Eosin, magnification x4. **(B)** Hemalun Eosin, magnification x 10.

A first vaginal biopsy under local anesthesia produced two small fragments of endometrial tissue with no atypia or malignancy (Figure 2). The biopsies did not contain any other tissue and no vaginal epithelium was found. Given the small size of the samples, new biopsies were suggested.

**Figure 3:**
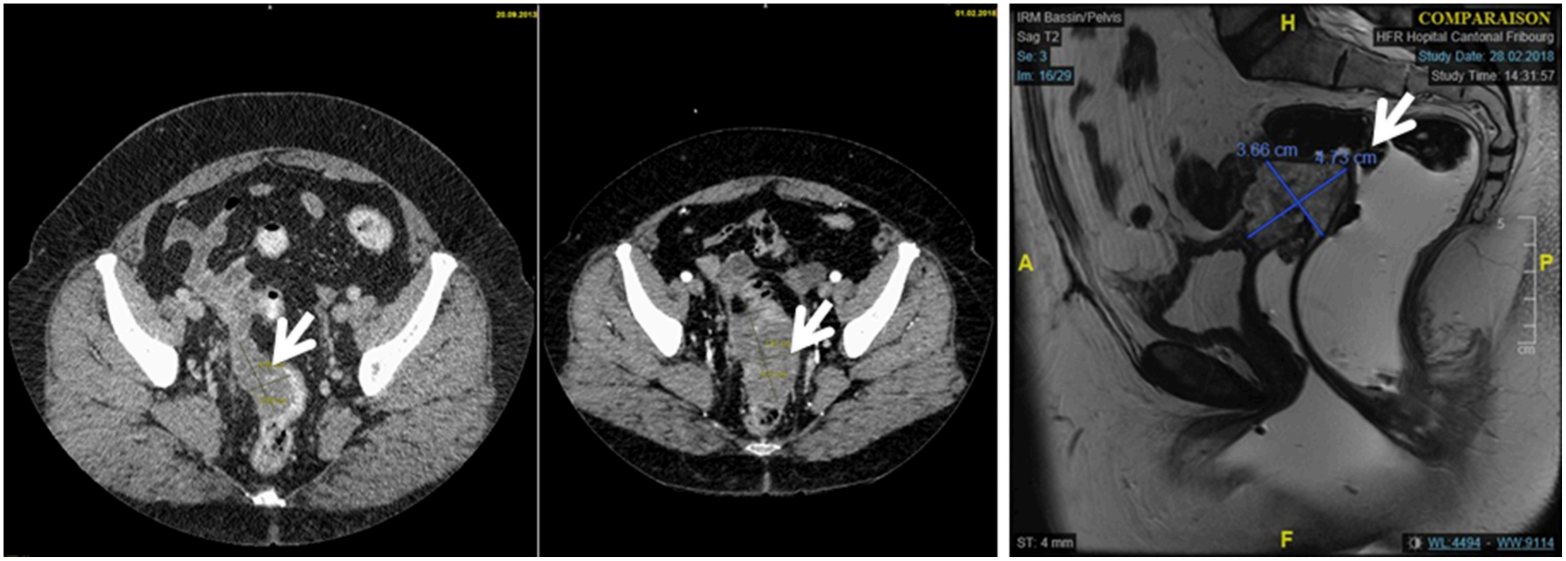
Comparison of the 2013 and 2018 CT scanner showing an enlargement of the vaginal mass from 48 x 24 mm to 76 x 47 mm.

**Figure 4:**
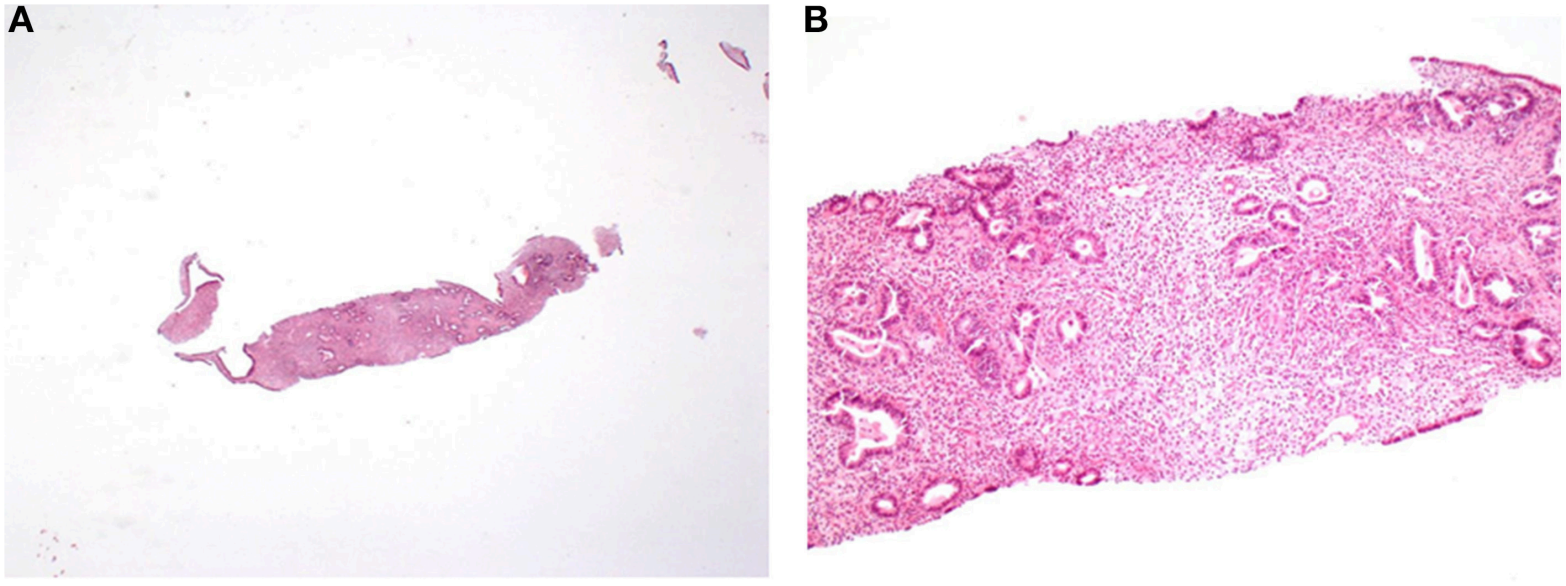
CT-scann guided core biopsies of a pelvic mass. This second series of biopsies again samples endometrium with a slightly denser stroma composed of fusocellular cells with rare mitotic figures and no significant atypia. **(A)** Hemalun Eosin, magnification x4. **(B)** Hemalun Eosin, magnification x 10.

The abdominal CT scan and MRI found a hypodense mass adjoining the vaginal dome possibly infiltrating the posterior bladder wall and the rectum (Figure 3). The recto-sigmoidoscopy was normal. A PET CT showed a high vaginal uptake of 18F-FDG and no lymphadenopathy or metastasis. The CT scan-guided biopsy produced small pieces of endometrium with limited stromal changes (mild stroma hyper-cellularity, with no significant atypia and very rare mitotic figures), with no evidence of a malignant tumor (Figure 4). The final pathological diagnosis was: endometrium without malignant features.

However, multidisciplinary discussions between gynecologists and pathologists, attempting to correlate a mass of unclear location (“vaginal”) with histological findings, lead to the potential hypothesis of a mullerian adenofibroma, a rare low grade neoplasm of the gynecological tract. It associates benign epithelial mullerian glands with a stromal component with few atypia (close to the endometrial stroma) and would be difficult to distinguish on small samples from its counterpart the low grade mullerian adenosarcoma. The results of immunohistochemical techniques, using antibodies against CD10, estrogen receptors, and epithelial markers, though not specific, were consistent with the mullerian adenofibroma hypothesis. Endometriosis was discussed but not retained because of the notion of hysterectomy in this patient.

Surgery was decided after multidisciplinary discussion. Before the surgery, a double J probe was inserted in ureters. Initially, a diagnostic laparoscopy was done, in conjunction with the visceral surgeons, showing a diffuse adherential status resolved by adhesiolysis. Bilateral adnexectomy by laparoscopy followed by a laparotomy conversion in order to extract the whole tumor. The coagulation and removal of the mass pedicle resulted in a bloc resection of the infiltrated recto-sigmoid with a small part of the vagina. A discharge ileostomy was performed. The operation lasted 8 h and the bleeding amounted to about 850 cc.

There were no immediate postoperative complication. Two weeks after the surgery, the patient developed a febrile state with a painful renal percussion and inflammatory syndrome with acute renal failure. After introducing antibiotic for pyelonephritis, the patient underwent a CT scan that showed a displaced right JJ probe that was removed the following day. The renal insufficiency was probably multifactorial and due to a context of infection, ileostomy liquid loss, toxicity of the antihypertensive drug, and acute tubular necrosis.

The evolution was favorable. The vaginal bleeding stopped after surgery. The ileostomy was closed 3 months later.

The pathological analysis of the 75 mm large, highly vascularized, rectosigmoid mass that had developed in the sigmoid meso and the *muscularis propria* concluded to endometriosis. Both ovaries harbored serous cystadenofibromas (left ovary: 2.7 cm long axis, right ovary: 2.5 cm long axis). No malignant neoplasm was found. No vaginal mucosa/wall was identified within the surgical specimen.

After phone call with the patient February 2019, she does not report any abdominal pain of vaginal bleeding. The last imagery and clinical control in Mai 2018 was normal.

## Discussion

Because of the complex symptomatology and the invasive manner of diagnosis, in the form of laparoscopy with visual findings of the endometriosis lesions (4), the exact prevalence of this pathology remains unknown. We suppose that an unknown but probably large portion of the population is asymptomatic. Pain is due to the inflammation-type reaction that gives rise to adhesion and distortion of physiological pelvic anatomy, but the degree of pain is not directly correlated to the severity of the disease (10).

Estrogenic stimulation by the ovary during the reproductive phase maintains endometriosis but, after menopause, several mechanisms are supposed to lead to the hormonal continuance which are hormone replacement therapy (HRT, without an associated progestin), and other estrogen secretors such as adipose tissue or the adrenal glands. Studies show that the endometriotic tissue could secrete its own estrogen, confirmed by the possible presence of aromatase expression in these ectopic lesions (11, 12). Research suggests that the postmenopausal state leads to a certain degree of immunosuppression that could perpetuate endometriosis (13), but it is not known whether it is a continuation of a past illness or a *de novo* development (14).

Although the physiological ovarian estrogenic secretion is over after menopause, this patient had several risk factors for maintaining a hyper estrogenic state, favorable to endometriosis, such as obesity and HRT (Estradiol, 4 years, stopped in 2018 before surgery). Retrospective anamnesis, the patient did not report any pain or fertility issues and the diagnosis of endometriosis was never assumed beforehand.

The first choice of treatment of postmenopausal endometriosis is a surgical procedure with optimal cytoreduction, by carbon dioxide ablation, laser, or bipolar diathermy (15), due to the risk of malignant degeneration (16). Until now, there has been no consensus on the most effective surgical technique to cure peritoneal disease and prevent recurrence (17). Studies show that a patient treated with ablation or excision of endometrioid lesion show a reduction of symptoms such as dyspareunia and pelvic pain (18).

The association of endometriosis with an increased risk of malignancies has been described (19) but is debated in the medical literature. Indeed, women with endometriosis were more likely to develop ovarian cancer than healthy ones (20), but the causality link is not clearly established. Most of the time, malignant transformation of endometriosis is correlated to endometrioids or clear cell carcinoma of the ovary (7). The primary remaining question is the frequency of the malignant transformation and, thus far, evidence is not strong enough. In 2008, Kobayashi et al. estimated the risk of malignant transformation for patient who suffer from pelvic endometriosis of 1% (21).

Age is a risk factor for many malignancies, from which we may hypothesize a higher malignant transformation potential risk for postmenopausal women with endometriosis (22).

For the moment, there are no recommendation for “prophylactic surgery” in women with endometriosis because of the malignant risk (8). Published in 2019, Kobayashi et al. made a literature review about the advances in the imaging and the non-invasive tests and biomarkers of early detection of the malignant transformation of benign ovarian endometrioma to endometriosis-associated ovarian cancer (23). Several techniques are investigated such as electronic absorption spectroscopy of cyst fluid hemoglobin or dosage of serum Tissue Factor Pathway Inhibitor-2 (TFPI2) (elevated with malignant transformation) (24).

Assuming a low-grade malignant tumor by viewing this mass, and the bleeding symptomatology, we performed surgery, but advanced endometriosis can be the cause of error or incorrect intraoperative evaluation by infiltrating the parametrium or lymphatic nodes or invasive procedures with complications (25).

Histological findings of the CT scan biopsy suggest a consistent with adenofibroma/sarcoma of low-grade diagnosis. But we know that the diagnosis of sarcoma is very difficult because of the histological and molecular heterogeneity and the rareness of this disease (26). Advances in management of sarcomas are made by implementing molecular assays and could help orienting the diagnostic (27).

Estrogenic dependency of endometriosis lesions explain the therapeutic potential of aromatase inhibitors that block the conversion from androgen to estrogen. This medication showed value in patients who are not eligible for surgery (28, 29) but this is not primarily recommended (30). Side effects with menopausal patients such as osteoporosis and fractures, hot flushes and vaginal dryness are to be considered before introducing such a treatment. If surgery is not an option, other painkiller medication is to be investigate, such as Desogestrel (progestin without estrogen). A recent study showed a reduction of pelvic endometriosis related symptoms (pain, dysmenorrhea) compared to a placebo group (31).

## Conclusion

It should be kept in mind that endometriosis is still an evocable diagnosis even after menopause and even after hysterectomy. The exact prevalence of endometriosis is still unknown because of the pauci-symptomatic nature and the invasiveness of the diagnostic procedure. Endometriotic lesions can mimic malignant lesions by their infiltrative character and produce a large panel of symptoms dependent on the site of the damage. Endometriosis is associated with cancer types such as endometrioid carcinoma of the ovary, but without proof of a causal link. The main procedure for treatment is surgery but should be balanced with the complication risks of a surgical procedure.

## Consent

Consent was obtained from the patient for the publication of this case report.

## Author Contributions

All authors listed have made a substantial, direct and intellectual contribution to the work, and approved it for publication.

### Conflict of Interest Statement

The authors declare that the research was conducted in the absence of any commercial or financial relationships that could be construed as a potential conflict of interest.
